# Does cycle commuting reduce the risk of mental ill-health? An instrumental variable analysis using distance to nearest cycle path

**DOI:** 10.1093/ije/dyad153

**Published:** 2024-01-15

**Authors:** Laurie Berrie, Zhiqiang Feng, David Rice, Tom Clemens, Lee Williamson, Chris Dibben

**Affiliations:** School of GeoSciences, University of Edinburgh, Edinburgh, UK; Scottish Centre for Administrative Data Research, University of Edinburgh, Edinburgh, UK; School of GeoSciences, University of Edinburgh, Edinburgh, UK; Scottish Centre for Administrative Data Research, University of Edinburgh, Edinburgh, UK; National Records of Scotland, Scotland’s Census—Geography, Edinburgh, UK; School of GeoSciences, University of Edinburgh, Edinburgh, UK; School of GeoSciences, University of Edinburgh, Edinburgh, UK; Scottish Centre for Administrative Data Research, University of Edinburgh, Edinburgh, UK; School of GeoSciences, University of Edinburgh, Edinburgh, UK; Scottish Centre for Administrative Data Research, University of Edinburgh, Edinburgh, UK

**Keywords:** Commuting, cycling, cycle commuting, active travel, mental health, instrumental variable analysis

## Abstract

**Background:**

Previous studies have linked cycling with improved mental wellbeing but these studies tend to use cross-sectional survey data that have small sample sizes and self-reported health measures, and are potentially susceptible to omitted-variable bias and reverse causation. We use an instrumental variable approach and an objective measure of mental ill-health taken from linked administrative data to ask: ‘Does cycle commuting reduce the risk of mental ill-health?’

**Methods:**

Our study links data on commuting in Edinburgh and Glasgow from the Scottish population census with mental health prescriptions from the National Health Service Prescribing Information System records. We use road distance from home to nearest cycle path as an instrumental variable for cycle commuting.

**Results:**

In total, 378 253 people aged 16–74 years living and working in the City of Edinburgh and Glasgow City council areas at the 2011 census were included in our study; 1.85% of commuters in Glasgow and 4.8% of commuters in Edinburgh cycled to work. Amongst cyclists, 9% had a prescription for mental health compared with 14% amongst non-cyclists. Using a bivariate probit model, we estimate a mean average reduction in prescriptions for antidepressants and/or anxiolytics in the 5 years following the census of –15.1% (95% CI: –15.3% to –15.0%) amongst cycle commuters compared with those who use any other mode to commute.

**Conclusions:**

This work suggests that cycle commuting is causally related to reduced mental ill-health and provides further evidence in support of the promotion of active travel to encourage commuters travelling shorter distances to shift to cycle commutes.

Key Messages

- Only a small percentage of the population of Scotland cycle to work.
- Research has shown that cycle commuting is beneficial to mental wellbeing.
- Using an instrumental variable analysis, we show that cycle commuting reduces the chances of being prescribed antidepressants and/or anxiolytics.
- This work provides further support for the expansion of cycling facilities and the promotion of active travel as an effective health intervention.

## Introduction

The positive effect of physical activity on mental health is well established^1^^,^^2^ with medium reductions in depression and small reductions in anxiety reported.^3^ However, the domain (e.g. sport, leisure, commuting) in which physical activity happens may influence its impact on mental health.^4^ Active commuting can be an important way for individuals to increase or maintain their physical activity levels as they are more likely to sustain physical activity levels if they can be incorporated into daily routines.^5^^,^^6^ Individual studies have linked cycle commuting to reduced all-cause mortality risk,^7–9^ decreased risk of cardiovascular disease^8–11^ and cancer-related mortality,^8^^,^^9^ and a systematic review reported the health benefits of cycling.^12^ In terms of mental health, cycle commuting has been linked to better subjective wellbeing,^13^^,^^14^ self-reported health^5^^,^^15^ and health-related quality of life.^16^ Long bicycle commutes are reported to be mentally relaxing and beneficial in the transition between work and home;^17^ however, a systematic review found the relationship between active commuting and depression to be inconsistent.^18^

It is conservatively estimated that poor mental health costs the Scottish economy £8.8 billion per year, with most of this cost being due to lost productivity amongst those living with mental health conditions. This does not include personal costs, such as those related to discrimination, social exclusion and stigmatization or absenteeism in the workplace. Approximately 40% of total costs can be attributed to major depression and anxiety disorders.^19^

Much research in this area has used small-scale observational data with the associated risks of omitted-variable bias and reverse causation. Infrastructure supporting the linkage of census and individual health records and the objective measure of mental health from prescription records provides us with the opportunity to improve on these previous studies.^13^^,^^14^ We use a pseudo experimental approach (using an instrumental variable) and linked administrative data comprising the whole population and their prescription data to ask: ‘Does cycle commuting reduce the risk of mental ill-health?’

## Methods

### Data source

We used data from the 2011 Scottish Census (census) linked to individual records from the Scottish National Prescription Information System (PIS) for the 5 years following the census date. The PIS covers all National Health Service (NHS) Scotland prescriptions, prescribed, dispensed and reimbursed in the community setting.^20^ The data were provided in anonymized format and analyses undertaken in a secure and regulated setting overseen by the electronic Data Research and Innovation Service (eDRIS), Public Health Scotland. Results were assessed by using National Records of Scotland and eDRIS prior to publication to ensure minimized risk of statistical disclosure of personal data.

### Study population

We restricted our study population to people living in Glasgow City and City of Edinburgh council areas, aged 16–74 years on census night who were in employment and commuted to their place of work. This equated to 400 814 people in 2011. We excluded those who had relevant prescriptions for mental ill-health (see ‘Outcome’) up to and including the month in which the census was held to capture occurrence rather than recurrence and those who lived >2 km from the nearest cycle path, as empirically we found that propensity to cycle was less well predicted at distances of >2 km (Supplementary Figure S1, available as Supplementary data at *IJE* online). Our final study population included 378 253 individuals.

### Variables

#### Exposure

The census asked: ‘How do you usually travel to your main place of work or study (including school)?’ Responses included the following: driving a car or van; passenger in a car or van; on foot; bus, minibus or coach; train; underground, subway, metro, light rail or tram; taxi or bicycle; motorcycle, scooter or moped; and other. We grouped these responses into a binary variable of bicycle vs all other modes of commute.

#### Outcome

Our outcome measure was new prescriptions for antidepressants (British National Formulary 4.3) and anxiolytics (British National Formulary 4.1.2) taken from the PIS data for prescriptions^21^ in the 5 years following the census. We did not include amitriptyline and nortriptyline at low doses (≤30 mg per day; i.e. three doses of the 10-mg tablet or one 25-mg tablet) from our antidepressant data set since these medicines are often used at low dose for non-mental illness-related conditions (e.g. neuropathic pain).^22^ From these data, we created a binary variable in which 0 was no prescription for antidepressants and/or anxiolytics in the time period and 1 was having any prescription for antidepressants and/or anxiolytics. Antidepressants are prescribed conservatively in Scotland and therefore there is good reason to believe the recipient of the prescription has been experiencing depression or anxiety.^23^ We therefore take a single prescription as an indication of mental ill-health.

#### Instrument

We use distance to a cycle path as our instrumental variable (IV)—this was calculated as the straight-line distance from an individual’s residential address to the nearest cycle path. Proximity has been used as an effective instrumental variable in other studies.^24^ The individual residential addresses are represented using grid references for the centroids of postcodes (average 30 households). The cycle paths for Edinburgh are from 2010 whereas the equivalent data for Glasgow are from 2014. The Feature Manipulation Engine was used to calculate the distance between residential postcode and nearest cycle path. The distance is the measure from the census postcode grid reference point to the node along the cycle path that is closest.

### Instrumental variable assumptions

A generalized linear model could potentially be biased were we to directly estimate the effect of cycling to work on mental health prescriptions due to unobserved confounding. This unobserved confounding could result in the error term of the model being correlated with the explanatory variables of the cycling-to-work variable. This means that the estimated effect of cycling to work would include the true effect of cycle commuting on prescriptions for mental health conditions but it would also capture the effect of the unobserved variable. To avoid this potential problem, we use an IV approach. IV analyses mimic randomized–controlled experiments in which the instrument effectively randomizes individuals to the exposure. To employ an IV analysis, we make the following assumptions: relevance, exchangeability and exclusion restriction.^25^

#### Relevance

For the instrument to be relevant, it must be associated with the exposure. A priori we note that there is evidence for this relationship.^26^^,^^27^ We also empirically test this assumption by calculating the F-statistic for the strength of the relationship between distance to cycle path and cycling.

#### Exchangeability

**Figure 1.**
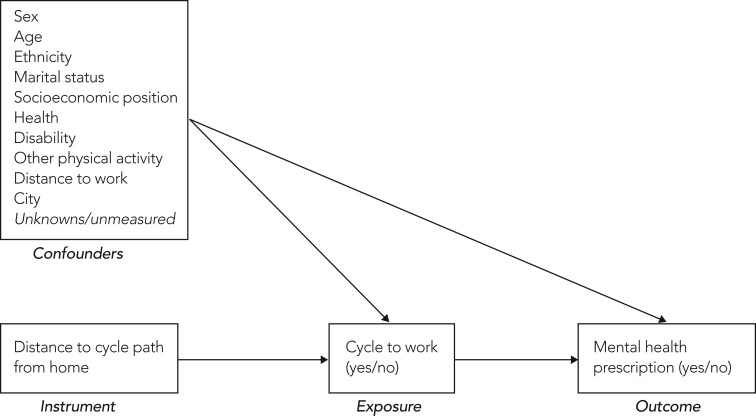
Causal diagram showing our instrument (distance to cycle path from home), exposure (whether individual cycles to work or uses another form of transport), outcome (whether individual has a mental health prescription) and confounders. Our list of confounders is for illustration and is not complete, as instrumental variable analysis does not require the strong assumption of no unmeasured confounding for the exposure and outcome

The exchangeability assumption requires that no common causes are shared between the instrument and the outcome. In the context of our study, this means that there are no common causes between the distance from home to the cycle path and whether an individual has a prescription for antidepressants or anxiolytics. Reviewing the literature, we find no evidence that there are any such common causes; therefore, we do not statistically adjust our analysis for other covariates (Figure 1). However, due to the systematic differences in cycling uptake and antidepressant and anxiolytic prescriptions between males and females, we run additional analyses separated by sex.

#### Exclusion restriction

Exclusion restriction requires that there is no relationship between the instrument and the outcome other than through the exposure. For our study, this means that the distance from home to the cycle path must not directly affect whether an individual has a prescription for antidepressants or anxiolytics. Given the development of the cycle path network to different parts of Edinburgh and Glasgow and the multiplicity of drivers of residential location decision-making, we feel it unlikely that there is a pathway through which distance from home to the cycle path affects whether an individual has a prescription for antidepressants or anxiolytics except via influencing whether the individual cycles to work or not.

### Analyses

We had a continuous instrument and binary exposure and outcome variables and used a bivariate probit (biprobit) model for our IV analysis. The biprobit model uses simultaneous likelihood estimation to estimate the average treatment effect.

All analyses were carried out in R^28^ except for the biprobit modelling, which was performed in Stata;^29^ 95% CIs were calculated using the Delta Method using the Stata ‘margins’ command.

### Sensitivity analyses

We also conduct a series of sensitivity analyses.

First, as it has been reported that physical activity reduces depression by a ‘medium’ amount and anxiety by a ‘small’ amount in non-clinical populations,^3^ we also model the prescriptions for antidepressants and anxiolytics separately, but not mutually exclusively. This also accounts for the differences in prescribing patterns between antidepressants and anxiolytics.

We remove those who walk to work from the data set, thereby comparing mental health between those who cycle and those who take part in non-active commuting as their main mode of commute.

We use a negative control outcome as suggested by Davies *et al.* to evaluate whether the IV assumptions hold.^30^ To do this, we replace prescriptions for antidepressants and anxiolytics with prescriptions for antipsychotics (British National Formulary 4.2)^21^ as our outcome of interest and limit this to prescriptions that occurred in our data set before the census took place. We believe this is a valid negative outcome control for our investigation because, in contrast to mood and anxiety disorders, genetic inheritance has a stronger impact on psychosis risk than environmental factors.^31^ Assuming that any confounders of the relationship between distance to cycle path and mental health are unlikely to be genetic in nature (and in particular jointly causing proximity to a cycle path and a heightened risk of schizophrenia) and that cycling to work is not particularly protective for genetically inherited schizophrenia, we would not expect a relationship between our IV and antipsychotic prescribing in the absence of an unknown causal factor or reverse causation affecting our study design. Consequently, no effect for antipsychotic prescribing in a replicated design would support the validity of our IV.

Finally, we carry out a ‘standard’ adjusted analysis using logistic regression to model the main outcome, prescriptions for anxiolytics and/or antidepressants and exposure, cycle commuting, whilst adjusting for measured confounders identified a priori with the aid of a Directed Acyclic Graph (Figure 1).

## Results

### Population characteristics

**Figure 2.**
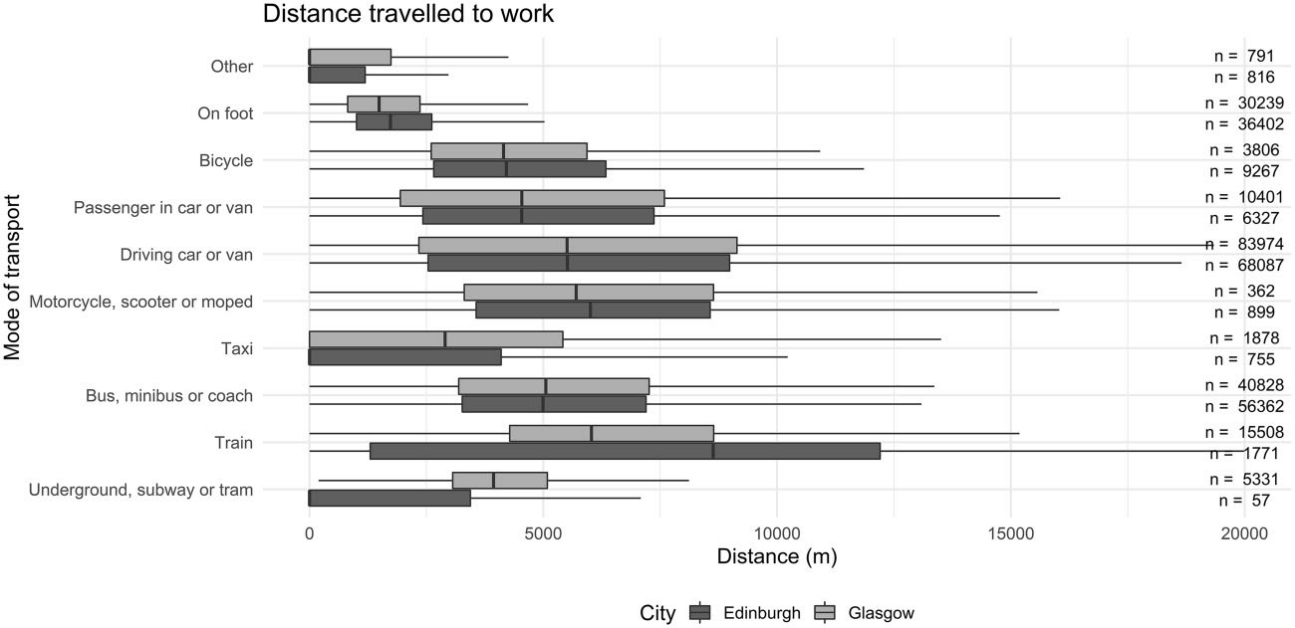
Distance travelled to work by people living in Glasgow City and City of Edinburgh council areas, aged 16–74 years on the 2011 census night who were in employment and commuted to their place of work

At the 2011 census, the percentage of commutes of <5 km for the City of Edinburgh and Glasgow City council areas were 54.4% and 51.8%, respectively. Of working people aged 16–74 years living in the Glasgow City council area, 1.85% cycled to work at the 2011 census (Figure 2). In the City of Edinburgh council area, this figure was 4.8%. Men were more likely to cycle to work than women. Of the 378 253 (males = 190 227, females = 188 026) in our study population, 9.1% of males and 15.6% of females had a prescription for anxiolytics or antidepressants; of those who cycled to work, 7.5% of males and 10.2% of females had a prescription for anxiolytics or antidepressants whereas for non-cyclists, 9.2% of males and 15.7% of females had a prescription for anxiolytics or antidepressants (Table 1). A further table is included in the Supplementary material (available as Supplementary data at *IJE* online) that shows population characteristics by prescription type (Supplementary Table S1, available as Supplementary data at *IJE* online).

**Table 1.** Characteristics of people living in Glasgow City and City of Edinburgh council areas, aged 16–74 years on the 2011 census night who were in employment and commuted to their place of work by whether they cycle to work or not

	Does not cycle to work		Cycles to work		Total	
Characteristic	Male (*n*=181 014)	Female (*n*=184 542)	Male (*n*=9213)	Female (*n*=3484)	Male (*n*=190 227)	Female (*n*=188 026)
Antidepressant or anxiolytic prescription						
No prescription	164 448 (90.8%)	155 485 (84.3%)	8526 (92.5%)	3128 (89.8%)	172 974 (90.9%)	158 613 (84.4%)
Prescription	16 566 (9.2%)	29 057 (15.7%)	687 (7.5%)	356 (10.2%)	17 253 (9.1%)	29 413 (15.6%)
Antidepressant prescription						
No prescription	169 645 (93.7%)	163 168 (88.4%)	8756 (95.0%)	3235 (92.9%)	178 401 (93.8%)	166 403 (88.5%)
Prescription	11 369 (6.3%)	21 374 (11.6%)	457 (5.0%)	249 (7.1%)	11 826 (6.2%)	21 623 (11.5%)
Anxiolytic prescription						
No prescription	173 119 (95.6%)	170 875 (92.6%)	8896 (96.6%)	3321 (95.3%)	182 015 (95.7%)	174 196 (92.6%)
Prescription	7895 (4.4%)	13 667 (7.4%)	317 (3.4%)	163 (4.7%)	8212 (4.3%)	13 830 (7.4%)
National Statistics Socio-economic classification						
Higher managerial, administrative and professional occupations	73 457 (40.6%)	79 144 (42.9%)	5629 (61.1%)	2267 (65.1%)	79 086 (41.6%)	81 411 (43.3%)
Intermediate occupations	35 086 (19.4%)	41 032 (22.2%)	1092 (11.9%)	481 (13.8%)	36 178 (19.0%)	41 513 (22.1%)
Routine and manual occupations	72 471 (40.0%)	64 366 (34.9%)	2492 (27.0%)	736 (21.1%)	74 963 (39.4%)	65 102 (34.6%)
Marital status						
Never married and never registered a same-sex civil partnership	88 635 (49.0%)	92 115 (49.9%)	4350 (47.2%)	1998 (57.3%)	92 985 (48.9%)	94 113 (50.1%)
Married or in a registered same-sex civil partnership	75 657 (41.8%)	67 572 (36.6%)	4264 (46.3%)	1197 (34.4%)	79 921 (42.0%)	68 769 (36.6%)
Separated but still legally married or in a civil partnership	5016 (2.8%)	6672 (3.6%)	163 (1.8%)	78 (2.2%)	5179 (2.7%)	6750 (3.6%)
Divorced or formerly in a civil partnership which is now dissolved	10 436 (5.8%)	14 937 (8.1%)	404 (4.4%)	196 (5.6%)	10 840 (5.7%)	15 133 (8.0%)
Widowed or surviving partner from a same-sex civil partnership	1270 (0.7%)	3246 (1.8%)	32 (0.3%)	15 (0.4%)	1302 (0.7%)	3261 (1.7%)
Ethnic group						
White	166 611 (92.0%)	174 248 (94.4%)	8893 (96.5%)	3360 (96.4%)	175 504 (92.3%)	177 608 (94.5%)
Mixed or multiple ethnic groups	809 (0.4%)	1055 (0.6%)	83 (0.9%)	37 (1.1%)	892 (0.5%)	1092 (0.6%)
Asian, Asian Scottish or Asian British	10 634 (5.9%)	7043 (3.8%)	149 (1.6%)	62 (1.8%)	10 783 (5.7%)	7105 (3.8%)
African	1718 (0.9%)	1435 (0.8%)	42 (0.5%)	11 (0.3%)	1760 (0.9%)	1446 (0.8%)
Caribbean or Black	308 (0.2%)	300 (0.2%)	17 (0.2%)	5 (0.1%)	325 (0.2%)	305 (0.2%)
Other ethnic group	934 (0.5%)	461 (0.2%)	29 (0.3%)	9 (0.3%)	963 (0.5%)	470 (0.3%)
Provision of unpaid care						
No	166 566 (92.0%)	163 565 (88.6%)	8574 (93.1%)	3199 (91.8%)	175 140 (92.1%)	166 764 (88.7%)
Yes	14 448 (8.0%)	20 977 (11.4%)	639 (6.9%)	285 (8.2%)	15 087 (7.9%)	21 262 (11.3%)
Self-reported general health						
Very good	107 548 (59.4%)	114 032 (61.8%)	6757 (73.3%)	2729 (78.3%)	114 305 (60.1%)	116 761 (62.1%)
Good	59 126 (32.7%)	57 545 (31.2%)	2146 (23.3%)	668 (19.2%)	61 272 (32.2%)	58 213 (31.0%)
Fair	12 017 (6.6%)	11 029 (6.0%)	261 (2.8%)	77 (2.2%)	12 278 (6.5%)	11 106 (5.9%)
Bad	1860 (1.0%)	1599 (0.9%)	41 (0.4%)	5 (0.1%)	1901 (1.0%)	1604 (0.9%)
Very bad	463 (0.3%)	337 (0.2%)	8 (0.1%)	5 (0.1%)	471 (0.2%)	342 (0.2%)
Self-reported long-term health problem or disability						
Yes, limited a lot	2981 (1.6%)	2828 (1.5%)	55 (0.6%)	15 (0.4%)	3036 (1.6%)	2843 (1.5%)
Yes, limited a little	8954 (4.9%)	9649 (5.2%)	318 (3.5%)	135 (3.9%)	9272 (4.9%)	9784 (5.2%)
No	169 079 (93.4%)	172 065 (93.2%)	8840 (96.0%)	3334 (95.7%)	177 919 (93.5%)	175 399 (93.3%)
City						
Edinburgh	85 008 (47.0%)	86 622 (46.9%)	6357 (69.0%)	2609 (74.9%)	91 365 (48.0%)	89 231 (47.5%)
Glasgow	96 006 (53.0%)	97 920 (53.1%)	2856 (31.0%)	875 (25.1%)	98 862 (52.0%)	98 795 (52.5%)
Age (years)						
Mean (SD)	39.2 (12.8)	38.4 (12.7)	38.5 (10.6)	37.2 (10.4)	39.2 (12.7)	38.4 (12.7)
Median [min, max]	38.0 [16.0, 74.0]	37.0 [16.0, 74.0]	38.0 [16.0, 74.0]	36.0 [16.0, 72.0]	38.0 [16.0, 74.0]	37.0 [16.0, 74.0]
Home to nearest cycle path (km)						
Mean (SD)	0.315 (0.283)	0.317 (0.283)	0.297 (0.250)	0.280 (0.221)	0.314 (0.282)	0.316 (0.282)
Median [min, max]	0.239 [0.0000203, 2.00]	0.241 [0.00002, 2.00]	0.236 [0.00034, 2.00]	0.224 [0.00002, 1.50]	0.239 [0.00002, 2.00]	0.241 [0.00002, 2.00]

### Instrument strength

The F-statistic, calculated from the logistic regression model, is 143. There is no ‘rule of thumb’ for the size of the F-statistic in the case in which the model is non-linear or with such a large sample size; we therefore use the recommendation from the literature for a cut-off of 16.38.^32^ This suggests that we do not have a weak instrument.^33^^,^^34^

### Biprobit model

The biprobit model estimates the average treatment effect of cycling to work on the outcome (i.e. prescriptions for antidepressants, anxiolytics or both) in the employed population of Edinburgh and Glasgow aged 16–74 years who commute to work. The average treatment effect is the difference in the mean average probability of the outcome occurring between those who cycle to work and those who do not. Here we find a –15.1% (95% CI: –15.3% to –15.0%) mean average reduction in the probability of receiving a prescription for antidepressants and/or anxiolytics in the 5 years following the census amongst cycle commuters compared with those who use any other mode of commute. All other results show a mean average reduction in mental health prescriptions in cycle commuters compared with those who use other modes of transport (Table 2) with this effect appearing to be smaller in males compared with females and amongst those with an anxiolytic prescription compared with an antidepressant prescription.

**Table 2.** Results of biprobit models estimating the average treatment effect of cycle commuting on each outcome

Data	Number	Outcome	Estimate (95% CI)
Male and female	378 253	Antidepressant and/or anxiolytic prescriptions	–15.1 (–15.3, –15.0)
Male and female	378 253	Antidepressant prescriptions	–11.8 (–11.9, –11.7)
Male and female	378 253	Anxiolytic prescriptions	–8.6 (–9.3, –8.0)
Female	188 026	Antidepressant and/or anxiolytic prescriptions	–17.1 (–17.4, –16.8)
Male	190 227	Antidepressant and/or anxiolytic prescriptions	–13.2 (–13.3, –13.0)
Female	188 026	Antidepressant prescriptions	–13.1 (–13.3, –12.8)
Male	190 227	Antidepressant prescriptions	–9.5 (–12.5, –6.6)
Female	188 026	Anxiolytic prescriptions	–8.9 (–9.5, –8.2)
Male	190 227	Anxiolytic prescriptions	–8.4 (–9.5, –7.3)
Male and female	378 253	Antipsychotics pre-census	0.000 (–0.003, 0.001)
Male and female	314 153	Antidepressant and/or anxiolytic prescriptions—walkers removed	–14.9 (–16.0, –13.8)

### Results of sensitivity analyses

The biprobit model removing those who walked to work as their main mode of transport from the data set estimates the average treatment effect as a –14.9% (95% CI: –16.0% to –13.8%) mean average reduction in the probability of receiving a prescription for antidepressants and/or anxiolytics in the 5 years following the census amongst cycle commuters compared with those who use any other ‘non-active’ mode of commute.

### Negative control outcome analysis

Modelling antipsychotics as our outcome of interest and limiting those occurring before the 2011 census, we estimated an average treatment effect of –0.000% (95% CI: –0.003% to 0.001%), suggesting that the distance from the place of residence to the nearest cycle path is not associated with potential confounders.

### Logistic regression analysis

Modelling prescriptions for antidepressants and/or anxiolytics as our outcome in a logistic regression model controlling for age, sex, ethnicity, marital status, the National Statistics Socio-economic Classification, city, self-reported health, whether they have a disability and whether a carer or not, we estimated a reduction in the relative odds of having a prescription of –13.4% (CI: –18.9% to –7.7%) for cycle commuters. This is distinct from the average treatment effects estimated using the biprobit models and therefore they are not directly comparable, although the direction and significance of the estimates are in agreement.

## Discussion

We found that cycling to work was causally associated with a decreased likelihood of having a prescription for antidepressants and/or anxiolytics in the 5 years following the point of travel measurement compared with those who used any other mode of commute. The size of the effect of cycle commuting on both anxiolytic and antidepressant prescriptions was greater for females than for males and greater amongst those who had antidepressant prescriptions compared with those who had anxiolytic prescriptions. These results and accompanying sensitivity analyses support existing research that found associations between cycle commuting and several other health domains.^12^

This is the first study to use distance from place of residence to nearest cycle path as an instrumental variable. The combination of the IV approach with the use of linked administrative data strengthens our conclusions as it allows a quasi-experimental design and full coverage of our population of interest (i.e. those in employment living in Glasgow City and City of Edinburgh council areas, aged 16–74 years on census night who were in employment and commuted to their place of work), respectively. By using both administrative data and the IV approach, we have mitigated the common shortcomings of previous studies including non-representative populations, omitted-variable bias and the limitations of subjective mental health measures. We also look at cycling separately to walking, which ensures that we are estimating the cycling-specific effects alone.

We rely on prescription data to measure mental ill-health as we did not have data on diagnosis for depression and anxiety; as such, our results may be influenced by the barriers to and stigma of accessing mental health treatment across different groups.^35^ Although this provided us with an objective measure, some of the medications of interest are also used for other conditions. To mitigate this as much as possible, we removed cases based on prescribed doses likely due to conditions other than depression and anxiety. Due to the probabilistic linkage of the administrative data sets used in this work, there will be some individuals whose data could not be linked and some groups may be more poorly linked than others; we have no reason to believe that linkage rates will be different between the exposed and unexposed groups, and linkage rates were high at 95+%.

We were unable to account for differences between general practitioners and their propensity to prescribe antidepressants and anxiolytics to treat depression and anxiety or diagnoses of depression and anxiety that were not subsequently treated with medication. These factors combined may mean that our total cases are lower than in reality, although the prevalence we report is <2%age points lower for women and <4%age points lower for women compared with those reported by the Scottish Public Health Observatory based on the Scottish Health Survey, as would be expected in this employed population.^36^ Additionally, Scottish prescription data are of high quality and are routinely collected and quality checked by NHS Scotland.^20^ It is unlikely that general practitioner prescribing practice will be associated with the patient’s home–cycle path distance, so it does not seem likely that this will have biased our analysis.

In order to capture the occurrence rather than recurrence of anxiety or depression, we excluded those who had relevant prescriptions for mental ill-health up to and including the month in which the census was held. As the PIS database only includes prescriptions from 2009 onwards, there will be some people who had prescriptions previous to this who will be captured as an occurrence rather than being omitted due to being a recurrence.

We do not have details of the frequency with which commuters use a bicycle to travel to work. We assume that the mode of commute a person reports in the census is the mode they usually use for their commute; however, the census does not capture whether a person uses more than one mode of commute, nor whether they commute only on certain days (e.g. part-time or hybrid working). For this reason, it is important to acknowledge that this research concerns the main mode of commute as (self-)reported at the census held in late March 2011, the timing of which may have influenced responses to the question regarding the commute mode.

There are several untestable assumptions that are necessary to use IV analysis. We use a negative control outcome analysis^30^ to evaluate whether our analysis is consistent with these assumptions and find support that distance to cycle path from place of residence is a relevant and strong IV. We also assume that distance from place of residence to nearest cycle path is random; we think this is an acceptable assumption. There are many factors that influence where a person chooses to live^37^ and, although being close to a cycle path may be one of those factors, we do not think it would override factors such as house price, type of housing stock available, local amenities or being close to friends and family. Cycle routes were being extended and added to in Glasgow and Edinburgh during the likely period of ownership/tenancy of our patients so for many, at the time of moving to a new residence, knowledge of the proximity of a future cycle path was impossible. Additionally, the cycle path data for Glasgow came from 2014 so some paths may be different from those present when the commute mode was recorded in the census.

The daily commute mode is influenced by a number of factors, such as weather,^38^ season, built environment, topography and meteorology. In this case, Edinburgh and Glasgow are different topographically and meteorologically, with Glasgow being the hillier and wetter of the two. A previous study has concluded that distance is probably the most important of these factors.^38^ It has been postulated that improved bicycle infrastructure will encourage cycling.^39^ An audit of bicycle infrastructure that featured Edinburgh as one of its six case study cities awarded Edinburgh the lowest bicycle infrastructure scores and reported the lowest governmental spend per person on cycling provision.^40^ The audit also makes recommendations for the considerations that should be made when designing bicycle infrastructure.

It is estimated that the annual health economic benefit of cycle commuting in Scotland at the 2011 census was EUR 79.8million.^41^ In terms of public health, the workplace can be an important setting in which to promote and protect mental health, and should be seen as an investment to employers rather than a cost.^19^ Recommendations for the organizational-level promotion of cycle commuting is beyond the scope of this article; however, recent work maps a broad range of actions that might be feasibly introduced and evaluated by employers (and others).^42^ Interventions should avoid widening health inequalities; it has been recognized elsewhere that cycling is gender and age unequal, and that increasing the number of cyclists will not automatically increase their diversity.^43^ A recent natural experimental study also concluded that the physical environment was more important for levels of cycling amongst men whilst the social environment was more complexly associated with cycling levels amongst women.^9^

We acknowledge that our study focuses on the population of commuters and that this population will be systematically different from the population not in work; therefore, our findings may not apply to those who are unemployed. Amongst the employed population, we recognize that not everyone would be able to cycle to work. However, there are many commuters for whom cycling would be an option and yet they choose to use another mode.^38^ There are a lot of commuters who travel <5 km using motorized transport^5^^,^^39^ that could be replaced by bicycle commutes. Our analysis has focused on two large cities; however, in rural areas (or for longer commutes starting or ending in cities), interventions could focus on public transport and links that include e-bikes and on-bus and on-train bicycle transport. Cycling to work could be a valuable way for individuals to attain recommended levels of physical activity.^12^

These findings could contribute support for increased investment in cycling infrastructure as there is evidence that increasing cycling infrastructure increases cycle commuting uptake.^44^^,^^45^ As well as the potential to improve mental health, this would reduce carbon emissions, congestion and air pollution when commuters move from non-active to active modes of travel as well as increase physical activity and create more liveable cities.^46^ Not all costs of mental health conditions are avoidable; however, interventions that could reduce or prevent a small fraction of them could be highly cost-effective.^19^

## Ethics approval

Approvals for this research were received from the ethics committee of the School of GeoSciences, University of Edinburgh, the Public Benefit and Privacy Panel for Health and Social Care (HSC-PBPP) and the Statistics Public Benefit and Privacy Panel.

## Supplementary Material

dyad153_Supplementary_Data

## Data Availability

This study uses sensitive patient data linked to the census. Access is restricted by law to approved researchers for use within the National Data Safe Havens only (the authors do not hold a copy of the data sets). Access is governed by the electronic Data Research and Innovation Service (eDRIS) of Public Health Scotland. Approved researchers can apply to eDRIS for data access, with further details available at https://www.isdscotland.org/Products-and-Services/EDRIS/. The variables required to replicate the analyses in this article are listed in the Supplementary material (available as Supplementary data at *IJE* online).

## References

[1] Cooney GM , Dwan K, Greig CA et al Exercise for depression. Cochrane Database Syst Rev 2013;2013:CD004366. https://www.cochranelibrary.com/cdsr/doi/10.1002/14651858.CD004366.pub6/full (16 October 2023; date last accessed) 24026850 10.1002/14651858.CD004366.pub6 PMC9721454

[2] Paluska SA , Schwenk TL. Physical activity and mental health. Sports Med 2000;29:167–80. 10739267 10.2165/00007256-200029030-00003

[3] Rebar AL , Stanton R, Geard D, Short C, Duncan MJ, Vandelanotte C. A meta-meta-analysis of the effect of physical activity on depression and anxiety in non-clinical adult populations. Health Psychol Rev 2015;9:366–78. 25739893 10.1080/17437199.2015.1022901

[4] White RL , Babic MJ, Parker PD, Lubans DR, Astell-Burt T, Lonsdale C. Domain-specific physical activity and mental health: a meta-analysis. Am J Prev Med 2017;52:653–66. 28153647 10.1016/j.amepre.2016.12.008

[5] M de H , Kroesen M, Chorus C, Hoogendoorn-Lanser S, Hoogendoorn S. Causal relations between body-mass index, self-rated health and active travel: an empirical study based on longitudinal data. J Transp Health 2021;22:101113.

[6] Chaix B , Kestens Y, Duncan S et al Active transportation and public transportation use to achieve physical activity recommendations? A combined GPS, accelerometer, and mobility survey study. Int J Behav Nutr Phys Act 2014;11:124. 25260793 10.1186/s12966-014-0124-x PMC4181295

[7] Shaw C , Blakely T, Atkinson J, Woodward A. Is mode of transport to work associated with mortality in the working-age population? Repeated census-cohort studies in New Zealand, 1996, 2001 and 2006. Int J Epidemiol 2020;49:477–85. 31930316 10.1093/ije/dyz257

[8] Celis-Morales CA , Lyall DM, Welsh P et al Association between active commuting and incident cardiovascular disease, cancer, and mortality: prospective cohort study. BMJ 2017;357:j1456. 28424154 10.1136/bmj.j1456

[9] Patterson R , Ogilvie D, Panter J. The social and physical workplace environment and commute mode: A natural experimental study. Prev Med Rep 2020;20:101260. 33318886 10.1016/j.pmedr.2020.101260 PMC7723790

[10] Munyombwe T , Lovelace R, Green M et al Association of prevalence of active transport to work and incidence of myocardial infarction: a nationwide ecological study. Eur J Prev Cardiol 2020;27:822–29. 31851832 10.1177/2047487319876228

[11] Eriksson JS , Ekblom B, Kallings LV et al Active commuting in Swedish workers between 1998 and 2015—Trends, characteristics, and cardiovascular disease risk. Scand J Med Sci Sports 2020;30:370–79. 31631386 10.1111/sms.13581 PMC7003943

[12] Oja P , Titze S, Bauman A et al Health benefits of cycling: a systematic review. Scand J Med Sci Sports 2011;21:496–509. 21496106 10.1111/j.1600-0838.2011.01299.x

[13] Mytton OT , Panter J, Ogilvie D. Longitudinal associations of active commuting with wellbeing and sickness absence. Prev Med 2016;84:19–26. 26740344 10.1016/j.ypmed.2015.12.010 PMC4766368

[14] Liu J , Ettema D, Helbich M. Systematic review of the association between commuting, subjective wellbeing and mental health. Travel Behav Soc 2022;28:59–74.

[15] Synek S , Koenigstorfer J. Health effects from bicycle commuting to work: Insights from participants of the German company-bicycle leasing program. J Transp Health 2019;15:100619.

[16] Neumeier LM , Loidl M, Reich B et al Effects of active commuting on health-related quality of life and sickness-related absence. Scand J Med Sci Sports 2020;30(Suppl 1):31–40. 10.1111/sms.13667 32246792

[17] Hansen KB , Nielsen TAS. Exploring characteristics and motives of long distance commuter cyclists. Transp Policy 2014;35:57–63.

[18] Marques A , Peralta M, Henriques-Neto D, Frasquilho D, Rubio Gouveira É, Gomez-Baya D. Active commuting and depression symptoms in adults: a systematic review. Int J Environ Res Public Health 2020;17:1041. 32041331 10.3390/ijerph17031041 PMC7037710

[19] McDaid D , Park A-L. The Economic Case for Investing in the Prevention of Mental Health Conditions in the UK. London: Mental Health Foundation; 2022.

[20] Alvarez-Madrazo S , McTaggart S, Nangle C, Nicholson E, Bennie M. Data Resource Profile: the Scottish National Prescribing Information System (PIS). Int J Epidemiol 2016;45:714–715f. 27165758 10.1093/ije/dyw060 PMC5005947

[21] Joint Formulary Committee. *British National Formulary [Internet].* https://bnf.nice.org.uk/ (22 August 2023, date last accessed)

[22] Moore RA , Derry S, Aldington D, Cole P, Wiffen PJ. Amitriptyline for neuropathic pain in adults. Cochrane Database Syst Rev 2015;2015:CD008242. 26146793 10.1002/14651858.CD008242.pub3 PMC6447238

[23] Cameron IM , Lawton K, Reid IC. Appropriateness of antidepressant prescribing: an observational study in a Scottish primary-care setting. Br J Gen Pract 2009;59:644–49. 19761665 10.3399/bjgp09X454061 PMC2734353

[24] Baiocchi M , Cheng J, Small DS. Instrumental variable methods for causal inference. Stat Med 2014;33:2297–340. 24599889 10.1002/sim.6128 PMC4201653

[25] Lousdal ML. An introduction to instrumental variable assumptions, validation and estimation. Emerg Themes Epidemiol 2018;15:1. 29387137 10.1186/s12982-018-0069-7 PMC5776781

[26] Krizek KJ , Barnes G, Thompson K. Analyzing the effect of bicycle facilities on commute mode share over time. J Urban Plann Dev 2009;135:66–73.

[27] Buehler R , Dill J. Bikeway networks: a review of effects on cycling. Transp Rev 2016;36:9–27.

[28] R Core Team. R: A Language and Environment for Statistical Computing. Vienna, Austria: R Foundation for Statistical Computing; 2022. https://www.R-project.org/ (22 August 2023, date last accessed)

[29] StataCorp. Stata Statistical Software: Release 16. College Station, TX: StataCorp LLC; 2019.

[30] Davies NM , Thomas KH, Taylor AE et al How to compare instrumental variable and conventional regression analyses using negative controls and bias plots. Int J Epidemiol 2017;46:2067–77. 28398582 10.1093/ije/dyx014 PMC5837536

[31] Picchioni MM , Murray RM. Schizophrenia. BMJ 2007;335:91–95. 17626963 10.1136/bmj.39227.616447.BE PMC1914490

[32] Stock J , Yogo M. Testing for weak instruments in linear IV regression. In: Andrews DWK, ed. Identification and Inference for Econometric Models. New York: Cambridge University Press; 2005, pp. 80–108. http://www.economics.harvard.edu/faculty/stock/files/TestingWeakInstr_Stock\%2BYogo.pdf (22 August 2023, date last accessed).

[33] Nichols A. *Causal Inference for Binary Regression with Observational Data*. Stata Users Group; 2011. https://www.stata.com/meeting/chicago11/materials/chi11_nichols.pdf (16 October 2023, date last accessed).

[34] Wooldridge J. *ivprobit Test of Instrument Strength*. www.statalist.org. 2014. https://www.statalist.org/forums/forum/general-stata-discussion/general/361582-ivprobit-test-of-instrument-strength (16 October 2023, date last accessed)

[35] Oliver MI , Pearson N, Coe N, Gunnell D. Help-seeking behaviour in men and women with common mental health problems: cross-sectional study. Br J Psychiatry 2005;186:297–301. 15802685 10.1192/bjp.186.4.297

[36] McLean J , Christie S, Gray L. *Scottish Health Survey 2016: Volume 1: Main Report*. The Scottish Government Health Directorate; 2017. https://www.gov.scot/publications/scottish-health-survey-2016-volume-1-main-report/ (6 November 2023, date last accessed).

[37] Thomas E , Serwicka I, Swinney P. *Urban Demographics Why People Live Where They Do*. 2015. https://www.centreforcities.org/wp-content/uploads/2015/11/15-11-02-Urban-Demographics.pdf (22 August 2023, date last accessed)

[38] Heinen E , van WB, Maat K. Commuting by bicycle: an overview of the literature. Transp Rev 2010;30:59–96.

[39] Gössling S , McRae S. Subjectively safe cycling infrastructure: new insights for urban designs. J Transp Geogr 2022;101:103340.

[40] Hull A , O’Holleran C. Bicycle infrastructure: can good design encourage cycling? Urban Plan Transp Res 2014;2:369–406.

[41] Baker G , Pillinger R, Kelly P, Whyte B. Quantifying the health and economic benefits of active commuting in Scotland. J Transp Health 2021;22:101111.

[42] Kelly P , Williamson C, Baker G et al; Cycle Nation Project. Beyond cycle lanes and large-scale infrastructure: a scoping review of initiatives that groups and organisations can implement to promote cycling for the Cycle Nation Project. Br J Sports Med 2020;54:1405–15. 32269057 10.1136/bjsports-2019-101447 PMC7677468

[43] Aldred R , Woodcock J, Goodman A. Does more cycling mean more diversity in cycling? Transp Rev 2016; 36:28–44.

[44] Yang Y , Wu X, Zhou P, Gou Z, Lu Y. Towards a cycling-friendly city: An updated review of the associations between built environment and cycling behaviors (2007–2017). J Transp Health 2019;14:100613.

[45] Mölenberg FJM , Panter J, Burdorf A, van Lenthe FJ. A systematic review of the effect of infrastructural interventions to promote cycling: strengthening causal inference from observational data. Int J Behav Nutr Phys Act 2019;16:93. 31655609 10.1186/s12966-019-0850-1 PMC6815350

[46] Banerjee A , Łukawska M, Jensen AF, Haustein S. Facilitating bicycle commuting beyond short distances: insights from existing literature. Transp Rev 2022;42:526–50.

