# Supplementary material for: Does cycle commuting reduce the risk of mental ill-health? An instrumental variable analysis using distance to nearest cycle path
Source: Int J Epidemiol. 2024 Jan 15;53(1):dyad153. doi: 10.1093/ije/dyad153 (PMC10859133; doi:10.1093/ije/dyad153)

# Supplementary Material

## Variables required to replicate analyses in “Does cycle commuting reduce the risk of mental ill-health? An instrumental variable analysis using distance to nearest cycle path.”

### 2011 Scottish Census held by National Records of Scotland

| Age |
| --- |
| Sex |
| Ethnicity |
| Marital Status |
| National Statistics Socio-economic situation (NSSEC) |
| Council area |
| Distance travelled to work |
| General health |
| Disability |
| Whether is a carer |
| Method of travel to work |

### Prescribing Information System (PIS) held by National Health Service National Services Scotland (NHS NSS)

| Prescribing Information System Month and Year Medicine Prescribed |
| --- |
| Prescribing Information British National Formulary Item Code |
| Prescribing Information Approved Name |
| Prescribing Information Item Strength / Unit of Measurement (UOM) |
| Prescribed Quantity |
| Prescribing Information BNF Subsection Description |

### Variables used for Linkage (but not accessible by research team)

| Community Health Index Number |
| --- |
| Forename |
| Surname |
| Date of Birth |
| Address |
| NHS Number |
| Postcode |
| NHS Number |

## Table S1: Population Characteristics by Prescription

Table S1: Characteristics of the people living in Glasgow City and City of Edinburgh council areas, aged 16-74 years on 2011 census night who were in employment and commuted to their place of work by whether they had a prescription for antidepressants, anxiolytics or both. SD = standard deviation, min = minimum, max = maximum

|  | Antidepressants and Anxiolytics | | Antidepressants Only | | Anxiolytics Only | |  |
| --- | --- | --- | --- | --- | --- | --- | --- |
|  | No prescription (N=331587) | Prescription (N=46666) | No prescription (N=344804) | Prescription (N=33449) | No prescription (N=356211) | Prescription (N=22042) | Total (N=378253) |
| **Sex** |  |  |  |  |  |  |  |
| Male | 172974 (52.2%) | 17253 (37.0%) | 178401 (51.7%) | 11826 (35.4%) | 182015 (51.1%) | 8212 (37.3%) | 190227 (50.3%) |
| Female | 158613 (47.8%) | 29413 (63.0%) | 166403 (48.3%) | 21623 (64.6%) | 174196 (48.9%) | 13830 (62.7%) | 188026 (49.7%) |
| **National Statistics Socio-economic classification** |  |  |  |  |  |  |  |
| Higher managerial, administrative and professional occupations | 143668 (43.3%) | 16829 (36.1%) | 148859 (43.2%) | 11638 (34.8%) | 152314 (42.8%) | 8183 (37.1%) | 160497 (42.4%) |
| Intermediate occupations | 67102 (20.2%) | 10589 (22.7%) | 70089 (20.3%) | 7602 (22.7%) | 72707 (20.4%) | 4984 (22.6%) | 77691 (20.5%) |
| Routine and manual occupations | 120817 (36.4%) | 19248 (41.2%) | 125856 (36.5%) | 14209 (42.5%) | 131190 (36.8%) | 8875 (40.3%) | 140065 (37.0%) |
| **Marital status** |  |  |  |  |  |  |  |
| Never married and never registered a same-sex civil partnership | 165074 (49.8%) | 22024 (47.2%) | 170550 (49.5%) | 16548 (49.5%) | 177453 (49.8%) | 9645 (43.8%) | 187098 (49.5%) |
| Married or in a registered same-sex civil partnership | 131122 (39.5%) | 17568 (37.6%) | 136956 (39.7%) | 11734 (35.1%) | 139756 (39.2%) | 8934 (40.5%) | 148690 (39.3%) |
| Separated but still legally married or in a civil partnership | 9810 (3.0%) | 2119 (4.5%) | 10327 (3.0%) | 1602 (4.8%) | 10920 (3.1%) | 1009 (4.6%) | 11929 (3.2%) |
| Divorced or formerly in a civil partnership which is now dissolved | 21683 (6.5%) | 4290 (9.2%) | 22878 (6.6%) | 3095 (9.3%) | 23863 (6.7%) | 2110 (9.6%) | 25973 (6.9%) |
| Widowed or surviving partner from a same-sex civil partnership | 3898 (1.2%) | 665 (1.4%) | 4093 (1.2%) | 470 (1.4%) | 4219 (1.2%) | 344 (1.6%) | 4563 (1.2%) |
| **Ethnic group** |  |  |  |  |  |  |  |
| White | 308321 (93.0%) | 44791 (96.0%) | 320924 (93.1%) | 32188 (96.2%) | 331937 (93.2%) | 21175 (96.1%) | 353112 (93.4%) |
| Mixed or multiple ethnic groups | 1794 (0.5%) | 190 (0.4%) | 1848 (0.5%) | 136 (0.4%) | 1900 (0.5%) | 84 (0.4%) | 1984 (0.5%) |
| Asian, Asian Scottish or Asian British | 16596 (5.0%) | 1292 (2.8%) | 17030 (4.9%) | 858 (2.6%) | 17287 (4.9%) | 601 (2.7%) | 17888 (4.7%) |
| African | 3008 (0.9%) | 198 (0.4%) | 3071 (0.9%) | 135 (0.4%) | 3115 (0.9%) | 91 (0.4%) | 3206 (0.8%) |
| Caribbean or Black | 560 (0.2%) | 70 (0.2%) | 581 (0.2%) | 49 (0.1%) | 599 (0.2%) | 31 (0.1%) | 630 (0.2%) |
| Other ethnic group | 1308 (0.4%) | 125 (0.3%) | 1350 (0.4%) | 83 (0.2%) | 1373 (0.4%) | 60 (0.3%) | 1433 (0.4%) |
| **Provision of unpaid care** |  |  |  |  |  |  |  |
| No | 301159 (90.8%) | 40745 (87.3%) | 312718 (90.7%) | 29186 (87.3%) | 322770 (90.6%) | 19134 (86.8%) | 341904 (90.4%) |
| Yes | 30428 (9.2%) | 5921 (12.7%) | 32086 (9.3%) | 4263 (12.7%) | 33441 (9.4%) | 2908 (13.2%) | 36349 (9.6%) |
| **Self-reported general health** |  |  |  |  |  |  |  |
| Very good | 208003 (62.7%) | 23063 (49.4%) | 215093 (62.4%) | 15973 (47.8%) | 220156 (61.8%) | 10910 (49.5%) | 231066 (61.1%) |
| Good | 101776 (30.7%) | 17709 (37.9%) | 106591 (30.9%) | 12894 (38.5%) | 111122 (31.2%) | 8363 (37.9%) | 119485 (31.6%) |
| Fair | 18552 (5.6%) | 4832 (10.4%) | 19675 (5.7%) | 3709 (11.1%) | 21079 (5.9%) | 2305 (10.5%) | 23384 (6.2%) |
| Bad | 2616 (0.8%) | 889 (1.9%) | 2771 (0.8%) | 734 (2.2%) | 3123 (0.9%) | 382 (1.7%) | 3505 (0.9%) |
| Very bad | 640 (0.2%) | 173 (0.4%) | 674 (0.2%) | 139 (0.4%) | 731 (0.2%) | 82 (0.4%) | 813 (0.2%) |
| **Self-reported long-term health problem or disability** |  |  |  |  |  |  |  |
| Yes, limited a lot | 4523 (1.4%) | 1356 (2.9%) | 4805 (1.4%) | 1074 (3.2%) | 5258 (1.5%) | 621 (2.8%) | 5879 (1.6%) |
| Yes, limited a little | 15098 (4.6%) | 3958 (8.5%) | 16027 (4.6%) | 3029 (9.1%) | 17144 (4.8%) | 1912 (8.7%) | 19056 (5.0%) |
| No | 311966 (94.1%) | 41352 (88.6%) | 323972 (94.0%) | 29346 (87.7%) | 333809 (93.7%) | 19509 (88.5%) | 353318 (93.4%) |
| **City** |  |  |  |  |  |  |  |
| Edinburgh | 161390 (48.7%) | 19206 (41.2%) | 167162 (48.5%) | 13434 (40.2%) | 171502 (48.1%) | 9094 (41.3%) | 180596 (47.7%) |
| Glasgow | 170197 (51.3%) | 27460 (58.8%) | 177642 (51.5%) | 20015 (59.8%) | 184709 (51.9%) | 12948 (58.7%) | 197657 (52.3%) |
| **Age** |  |  |  |  |  |  |  |
| Mean (SD) | 38.7 (12.8) | 39.7 (12.2) | 38.8 (12.8) | 39.0 (12.2) | 38.7 (12.7) | 40.9 (12.1) | 38.8 (12.7) |
| Median [Min, Max] | 37.0 [16.0, 74.0] | 40.0 [16.0, 74.0] | 38.0 [16.0, 74.0] | 39.0 [16.0, 74.0] | 38.0 [16.0, 74.0] | 41.0 [16.0, 74.0] | 38.0 [16.0, 74.0] |
| **Home to nearest cycle path (km)** |  |  |  |  |  |  |  |
| Mean (SD) | 0.314 (0.281) | 0.325 (0.294) | 0.314 (0.281) | 0.324 (0.292) | 0.314 (0.281) | 0.326 (0.295) | 0.315 (0.282) |
| Median [Min, Max] | 0.239 [0.0000203, 2.00] | 0.246 [0.0000203, 2.00] | 0.239 [0.0000203, 2.00] | 0.246 [0.000340, 2.00] | 0.239 [0.0000203, 2.00] | 0.245 [0.0000203, 2.00] | 0.240 [0.0000203, 2.00] |

## Figure S1: Propensity to Cycle – Logistic Regression

We used a logistic regression to model the outcome cycle to work (yes = 1, no = 0) against the distance from home to cycle path (km) amongst a total population of 386 058 and found an odds ratio of 0.811 (95% confidence interval: 0.779 to 0.846). The termplot below shows that distance to cycle path predicts population cycle commute uptake well up to 2km, therefore, we remove the small number of people who live more than 2km from a cycle path (2%) resulting in a population of 378 253.


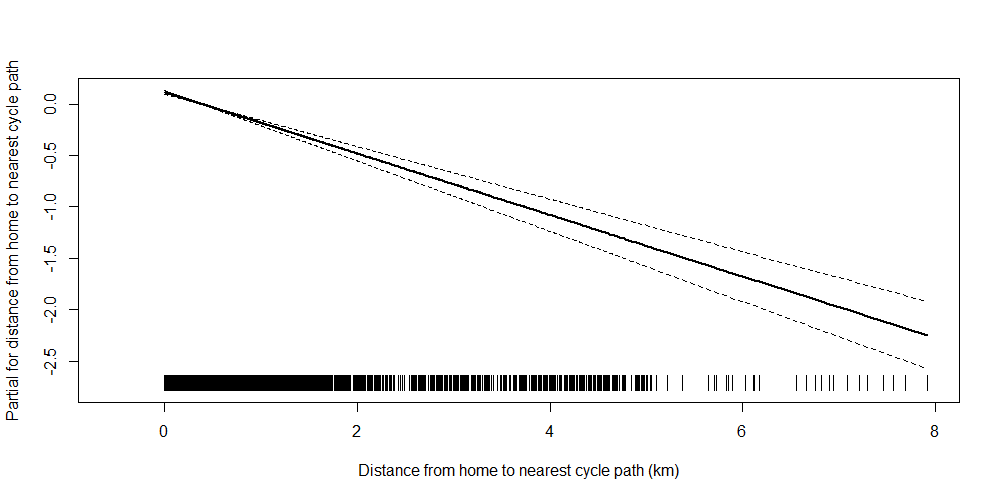

Supplement: dyad153_Supplementary_Data [file dyad153_supplementary_data.docx]
